# Dose Super-Resolution in Prostate Volumetric Modulated Arc Therapy Using Cascaded Deep Learning Networks

**DOI:** 10.3389/fonc.2020.593381

**Published:** 2020-11-16

**Authors:** Dong-Seok Shin, Kyeong-Hyeon Kim, Sang-Won Kang, Seong-Hee Kang, Jae-Sung Kim, Tae-Ho Kim, Dong-Su Kim, Woong Cho, Tae Suk Suh, Jin-Beom Chung

**Affiliations:** ^1^ Department of Biomedical Engineering, Department of Biomedicine and Health Sciences, College of Medicine, The Catholic University of Korea, Seoul, South Korea; ^2^ Research Institute of Biomedical Engineering, College of Medicine, The Catholic University of Korea, Seoul, South Korea; ^3^ Department of Radiation Oncology, Seoul National University Bundang Hospital, Bundang, South Korea; ^4^ Proton Therapy Center, National Cancer Center, Goyang, South Korea; ^5^ Korea Atomic Energy Research Institute, Daejeon, South Korea; ^6^ Department of Radiation Oncology, Seoul National University Boramae Medical Center, Seoul, South Korea

**Keywords:** deep learning, cascaded networks, dose super-resolution, dose grid size, prostate volumetric modulated arc therapy

## Abstract

**Purpose:**

This study proposes a cascaded network model for generating high-resolution doses (i.e., a 1 mm grid) from low-resolution doses (i.e., ≥3 mm grids) with reduced computation time.

**Methods:**

Using the anisotropic analytical algorithm with three grid sizes (1, 3, and 5 mm) and the Acuros XB algorithm with two grid sizes (1 and 3 mm), dose distributions were calculated for volumetric modulated arc therapy plans for 73 prostate cancer patients. Our cascaded network model consisted of a hierarchically densely connected U-net (HD U-net) and a residual dense network (RDN), which were trained separately following a two-dimensional slice-by-slice procedure. The first network (HD U-net) predicted the downsampled high-resolution dose (generated through bicubic downsampling of the baseline high-resolution dose) using the low-resolution dose; subsequently, the second network (RDN) predicted the high-resolution dose from the output of the first network. Further, the predicted high-resolution dose was converted to its absolute value. We quantified the network performance using the spatial/dosimetric parameters (dice similarity coefficient, mean dose, maximum dose, minimum dose, homogeneity index, conformity index, and V_95%_, V_70%_, V_50%_, and V_30%_) for the low-resolution and predicted high-resolution doses relative to the baseline high-resolution dose. Gamma analysis (between the baseline dose and the low-resolution dose/predicted high-resolution dose) was performed with a 2%/2 mm criterion and 10% threshold.

**Results:**

The average computation time to predict a high-resolution axial dose plane was <0.02 s. The dice similarity coefficient values for the predicted doses were closer to 1 when compared to those for the low-resolution doses. Most of the dosimetric parameters for the predicted doses agreed more closely with those for the baseline than for the low-resolution doses. In most of the parameters, no significant differences (p-value of >0.05) between the baseline and predicted doses were observed. The gamma passing rates for the predicted high-resolution does were higher than those for the low-resolution doses.

**Conclusion:**

The proposed model accurately predicted high-resolution doses for the same dose calculation algorithm. Our model uses only dose data as the input without additional data, which provides advantages of convenience to user over other dose super-resolution methods.

## Introduction

Volumetric modulated arc therapy (VMAT) delivers radiation doses, with variable dose rate, continuously *via* the dynamic movement of gantry/multileaf collimator (MLC) leaves, and is capable of delivering highly conformal prescription doses to the target while minimizing to the exposure for organs at risks (OARs) (1–4).The advantages of VMAT are that it has fewer monitor unit requirements and a shorter delivery time compared to intensity modulated radiation therapy (IMRT). Consequently, VMAT is the preferred technique in many clinics (5). Precise dose calculation is paramount to ensure accurate dose delivery using VMAT (6). In general, the dose calculation is performed using various algorithms, including collapsed cone convolution (CCC) (7), the anisotropic analytical algorithm (AAA) (8, 9), Acuros XB (AXB) (10, 11), and X-ray voxel Monte Calro (12), in commercial treatment planning systems (TPSs). Among these algorithms, the AAA and the AXB algorithm are widely utilized through the Eclipse TPS (Varian Medical Systems, Palo Alto, CA, USA).

Several previous studies have reported that dose grid size is related to the accuracy of dose calculations using the AAA and the AXB algorithms in VMAT/IMRT plans. (13-19) With film/Monte Carlo evaluations, Gagne et al. (13) showed that AAA dose calculations with a 5 mm grid caused >2% dosimetric error than those with finer grids (≤ 2.5 mm) in a simple RapidArc (a form of VMAT used by Varian Medical Systems) plan. Ong et al. (14) reported that dose differences relating to grid size (2.5 vs. 1 mm) for the AAA calculation were up to 20% and 5% for small MLC fields and a RapidArc plan, respectively. Kan et al. (15) demonstrated that using the AXB algorithm with a 1 mm grid improved dose accuracies (within 3%) as opposed to a 2.5 mm grid using verification of point doses in anthropomorphic phantom for a stereotactic IMRT plan. They recommended the use of a 1 mm grid in a stereotactic plan using a computed tomography (CT) image with 1.25 mm slice spacing. Akino et al. (16) showed that superficial doses (for a breast IMRT plan) calculated using the AAA with a 1 mm grid are closer to measured doses (by film) than those using a 2.5 mm grid. They demonstrated that, compared with a 2.5 mm grid, the use of a 1 mm grid improved dose underestimation from 19.1% to 12.0%. Through gamma evaluation of the IMRT plan for head and neck patients, Srivastava et al. (17) found that doses based on the AAA with a 1 mm grid showed closest agreement with the measured doses (by film) compared to AAA dose calculations for several grid sizes (2, 3, 4, and 5 mm). Their findings indicated that use of a 1 mm grid is essential for treating head and neck patients *via* the IMRT plan because of small OARs involved, for example, optic nerve and cochlea. Chow et al. (18, 19) showed there were variations of dose volume parameters and radiobiological parameters on planning target volume (PTV), rectal wall, and rectum according to changes of grid sizes (from 1 to 5 mm with 1 mm intervals) using AAA calculation in prostate VMAT plan.

As reported in aforementioned studies, a 1 mm grid facilitates precise dose calculation by reducing the volume averaging effect (20, 21), which becomes more pronounced when using larger grid sizes (>1 mm). Specially, larger grid sizes can lead to noticeable errors in highly modulated plans (14), stereotactic plans (15), surface/superficial regions (16), and small OAR structures (17). Furthermore, errors for radiobiological parameters (tumor control probability and normal tissue complication probability) can occur on several regions (PTV, rectum wall, and rectum) in prostate VMAT (18, 19). These errors may cause a clinically significant problem in above mentioned cases. Therefore, a 1 mm grid is the most suitable for performing accurate dose calculations.

Despite the dosimetric advantage associated with using a 1 mm grid, many clinics typically use 2–5 mm grids because of relatively long computation time for a 1 mm grid. We reported the computation times for the AAA and the AXB algorithm according to grid sizes in prostate VMAT plans (AAA with a 1 mm grid: 2,211 ± 155 s, AAA with a 3 mm grid: 245 ± 27 s, AAA with a 5 mm grid: 130 ± 10 s, AXB with a 1 mm grid: 4061 ± 922 s, AXB with a 2 mm grid: 671 ± 91 s, AXB with a 3 mm grid: 262 ± 26 s) in a previous study (22).

Recent advances in artificial intelligence technology such as machine learning (linear regression, regression tree, support vector machine, deep neural network, and etc.) have helped solve complex problems in radiotherapy (23), such as auto-segmentation (24, 25), dose prediction (26–28), synthetic CT image generation (29, 30), and prediction of treatment planning evaluation parameters (31) as well as support for time-consuming work like patient-specific quality assurance (32, 33). In general, these artificial intelligence models need a relatively long computation time to training. However, once they are learned, the computation time to solve a problem is very short. Because of this advantage, several groups have recently proposed a dose super-resolution method with reduced computation time using deep learning (34, 35). Dong and Xing (34) presented Deep DoseNet, which transformed the AAA-calculated dose with a 5 mm grid to that generated by AXB algorithm with a 1.25 mm grid. In their proposed network, downsampled CT and dose slices were used simultaneously as the inputs. The authors used beam dose distributions derived from artificially generated plans according to iso-center location, field size, and gantry angle rather than clinical plans. Alternatively, Sumida et al. (35) suggested a U-net based model, which used the AAA-calculated dose with a 5 mm grid and a CT image as the inputs, to predict the AXB-calculated dose with a 2 mm grid for applying prostate VMAT. Although these previously proposed deep learning networks predicted the high-resolution dose accurately, several caveats should be noted, including the use of simplified datasets based on beam dose distribution rather than clinical data (34) or the prediction of doses for a 2 mm grid only (35); a simplified dataset could lead to ideal results without clinical relevance, and doses calculated with a 2 mm grid are less accurate than those with a 1 mm grid.

To overcome these limitations, we propose a cascaded network model capable of generating high-resolution doses (with a 1 mm grid) from low-resolution doses (with ≥3 mm grids) with reduced computation time in prostate VMAT. This paper describes the details of network construction/data preparation (acquisition, preprocessing, data selection, and augmentation)/network training and quantifies the network performance by calculating the spatial/dosimetric parameters for the low-resolution and predicted high-resolution doses relative to the baseline high-resolution dose with a 1 mm grid.

## Materials and Methods

### Network Construction

**Figure 1:**
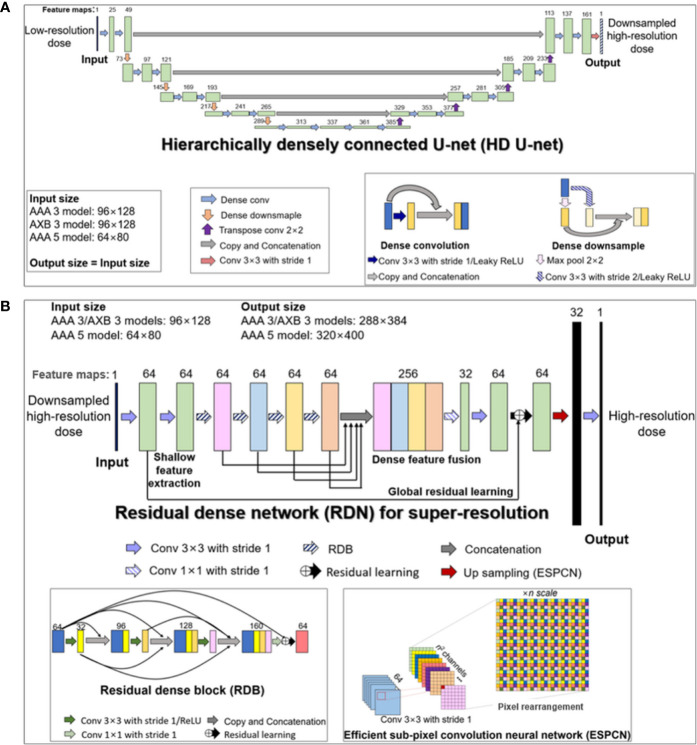
Structures of the two deep learning networks used in this study, which were connected in a cascaded manner: **(A)** modified hierarchically densely connected U-net (HD U-net); **(B)** residual dense network (RDN) for super-resolution based on sub-pixel convolution. The HD U-net used a low-resolution dose as an input, with the output of the HD U-net used subsequently as an input of the RDN. The numbers above the colored boxes indicate the number of feature maps. The anisotropic analytical algorithm (AAA) 3, AAA 5, and AXB 3 models indicate network models for ×3 super-resolution scale (predictions from 3 mm to 1 mm grid) in AAA and ×5 super-resolution scale (from 5 mm to 1 mm grid) in the AAA, and ×3 super-resolution scale in the AXB algorithm, respectively.

**Figure 2:**
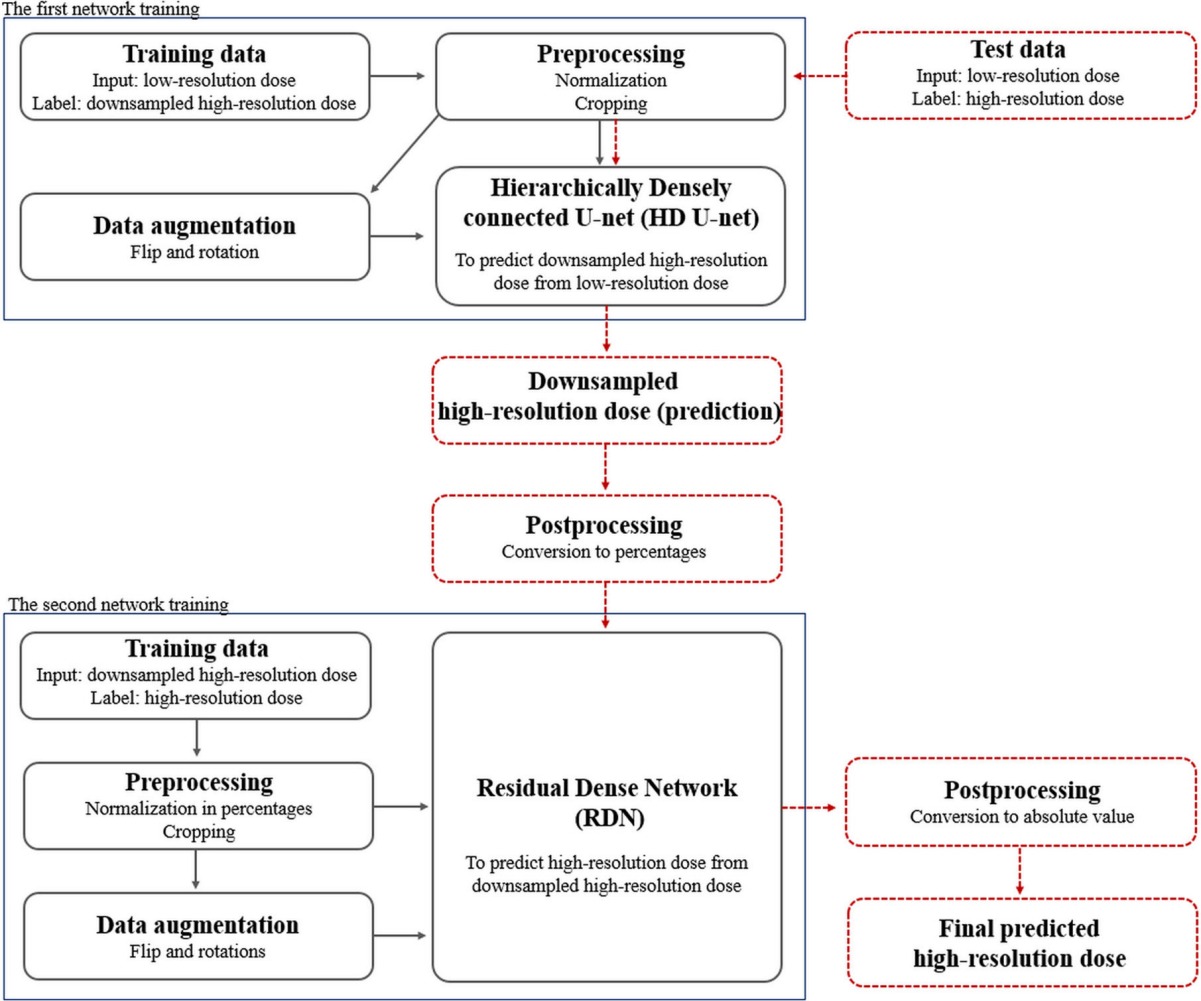
Schematic overview of the proposed dose super-resolution workflow for network training and testing. Training data were preprocessed with normalization/cropping and augmented using flip/rotations. Two networks (hierarchically densely connected U-net, HD U-net; residual dense network, RDN) are trained separately (solid black arrows), and then connected in a cascaded manner in testing (dashed red arrows). The first network (HD U-net) predicts the downsampled high-resolution dose plane using the low-resolution dose plane. The second network (RDN) predicts the high-resolution dose using output from the first network as its input. Finally, the predicted high-resolution dose was converted to its absolute value.

Our cascaded network model consisted of (a) a hierarchically densely connected U-net (HD U-net) (27), which utilizes both local and global information with efficient feature propagation/reuse, and (b) a residual dense network (RDN) (36), which is capable of using hierarchical features from the input data to their full extent, as shown in **Figure 1**. Each network was trained separately in a two-dimensional (2D) slice-by-slice procedure. The first network (HD U-net) predicted the downsampled high-resolution dose (generated through bicubic downsampling of the baseline high-resolution dose with a 1 mm grid) using the low-resolution dose with a large grid (≥3 mm). Then, the second network (RDN) predicted the high-resolution dose with a 1 mm grid using the output from the first network as its input. Finally, the predicted high-resolution dose was converted to its absolute value. **Figure 2** illustrates the schematic workflow for generating super-resolution doses using our network model. Our proposed networks predicted the high-resolution dose with a single super-resolution scale in the same dose calculation algorithm. We generated three models according to calculation algorithms and grid sizes: (a) AAA 5 model (predictions from AAA-calculated dose with a 5 mm grid to that with a 1 mm grid), (b) AAA 3 model (from AAA-calculated dose with a 3 mm grid to that with a 1 mm grid), and (c) AXB 3 model (from AXB-calculated dose with a 3 mm grid to that with a 1 mm grid).

#### Hierarchically Densely Connected U-Net (HD U-Net)

The HD U-net predicts the downsampled high-resolution dose (derived from the baseline dose with a 1 mm grid) using the low-resolution dose as the input. Therefore, the network was trained using low-resolution (input) and downsampled high-resolution dose (label) datasets. **Figure 1A** describes the modified HD U-net structure used in this study; a grow rate (defined as the number of added new feature maps *via* each convolution operation) and an activation function were modified to 24 and a leaky rectified linear unit (ReLU), respectively. In general, the original HD U-net (24) has a similar structure to the original U-net (37) except for two operations: (a) dense convolution and (b) dense downsampling. In the modified HD U-net used herein, the dense convolution included a 3×3 convolution (stride 1 and 24 filters) with a leaky ReLU and a concatenation with the previous feature map. The dense downsampling consisted of a 3×3 convolution (stride 2 and 24 filters) with a leaky ReLU, a 2×2 max pooling (stride 2), and a concatenation with the convolved and max-pooled feature maps. In the left side of the HD U-net, the dense convolution and downsampling operations were repeated 4 times at a ratio of 2:1. In the right side, the dense downsampling was replaced with a 2×2 transposed convolution with 64 filters for upsampling. Each upsampled feature map was concatenated with the corresponding feature map from the left side (known as skip connection). At the last layer, a 3×3 convolution (stride 1 and filter 1) without an activation function was used to map 161 features to 1 feature.

#### Residual Dense Network (RDN) for Super-Resolution

We used the RDN structure for image super-resolution proposed by Zhang et al. (36). The RDN used herein predicts the high-resolution dose with a 1 mm grid from the output of the HD U-net. To perform this prediction, we trained the RDN using the downsampled high-resolution dose (input) and the high-resolution dose (label). As shown in **Figure 1B**, the RDN consisted of a shallow feature extraction operation, residual dense blocks (RDBs), dense feature fusion, global residual learning, and an efficient sub-pixel convolution neural network (ESPCN) (38). The shallow feature extraction was performed first, using two 3×3 convolution layers (stride 1 and 64 filters) without an activation function. The RDB included densely connected layers (with each layer connected to other all layers in a feed-forward fashion), local feature fusion (a concatenation of feature maps from each layer, followed by 1×1 convolution) and local residual learning. The dense feature fusion was processed by a concatenation of feature maps from four RDBs, followed by 1×1 convolution (stride 1 and 32 filters). The global residual learning was performed *via* skip connection between the output of the first convolved layer and the output of the last layer (before upsampling). The ESPCN, which performed upsampling *via* rearrangement of the pixels of feature maps, was applied to the upsampling layer. At the last layer, a 3×3 convolution (stride 1 and filter 1) without activation function was used to map 32 features to 1 feature.

### Data Preparation

Data preparation consisted of acquisition, preprocessing (normalization and cropping), data selection, and augmentation steps. For the data acquisition, 73 patients who received prostate VMAT (prescription: 78 Gy/39 fractions for 20 patients and 70 Gy/28 fractions for 53 patients), were selected. For each patient, dose distributions were computed according to calculation algorithms and grid sizes under the same treatment plan; the high-resolution doses (with a 1 mm grid for both the AAA and the AXB algorithm) and low-resolution doses (with 3 and 5 mm grids for the AAA and a 3 mm grid for the AXB algorithm) were acquired. Subsequently, downsampled high-resolution doses were generated by bicubic interpolation of the high-resolution doses to a matrix size of the low-resolution doses. The datasets were split uniformly at random into training (80%) and test groups (20%). In preprocessing, the doses for test/training datasets were normalized according to the prescription dose to correct for bias deriving from different prescription doses (78 or 70 Gy). Then, cropping was used to remove unnecessary regions such as those outside of the body structure. In the data selection step, only axial dose planes having a pixel dose with >10% of the prescription (empirically selected value) were selected. This data selection process was not applied to the test dataset used to quantify the network performance. Finally, the training dataset was augmented using flip and rotations (from −15 to 15° with 5° intervals).

#### Data Acquisition

The Institutional Review Board (IRB) of our institute approved the data collection (Seoul National Bundang Hospital Protocol B-2004/608-112). Computed tomography images were acquired for 73 prostate cancer patients using a CT scanner (Brilliance CT Big Bore, Philips, Eindhoven, Netherlands). The pixel spacing of the acquired CT images was 1.17×1.17 mm^2^. These CT images were exported to Eclipse TPS (version 13.7.16, Varian Medical System, Palo Alto, CA, USA). Then, contours for PTV and OARs (rectum, bladder, and left/right femoral heads) were delineated by a radiation oncologist. Subsequently, prostate VMAT plans were generated with prescription doses of 78 Gy in 39 fractions (53 patients) or 70 Gy in 28 fractions (20 patients). The details of the plan information, including dose volume constraint, were described in our previous study (22). For each patient, the dose distributions were computed using the AAA and the AXB algorithm according to grid sizes under the same plans. In total, the high-resolution doses (with a 1 mm grid for the both the AAA and the AXB algorithm) and low-resolution doses (with 3 and 5 mm grids for the AAA and a 3 mm grid for the AXB algorithm) were obtained. To match the coordinates between the high-resolution doses and low-resolution doses, we rigidly registered (translation in left–right, superior–inferior, and anterior–posterior directions) the high-resolution doses to the coordinates of the low-resolution doses using Image Position and Orientation information on DICOM headers. Then, we downsampled the high-resolution doses to the matrix size of the low-resolution doses using bicubic interpolation. Finally, the dose datasets were split uniformly at random into training (80%) and test groups (20%). The distribution of the datasets can be seen in the **Supplementary Data**.

#### Preprocessing

The preprocessing step comprised normalization and cropping. First, the datasets were normalized according to the prescription dose to correct for bias derived from the two different prescriptions (i.e., 78 or 70 Gy). Cropping was used to remove irrelevant regions such as those outside of the body structure, and was performed to fit the following matrix sizes: (a) 96×128 for a 3 mm grid, (b) 64×80 for a 5 mm grid, (c) 288×384 for a 1 mm grid, and (d) 320×400 for a 1 mm grid. The 96×128 and 64×80 sizes indicated the matrix dimensions for axial dose planes using 3 and 5 mm grids, respectively. The other sizes represented the matrix dimensions of axial dose planes in the high-resolution doses. The 96×128 and 288×384 dose planes were paired together for training of the AAA 3/AXB 3 models (defined in *Materials and Methods*. A.). For training of the AAA 5 model, 64×80 dose planes were paired with 320×400 dose planes.

#### Data Selection and Augmentation

We selected only axial dose planes having a pixel dose >10% of the prescription (empirically selected value). This is because, for each patient, most dose planes did not include a sharp dose gradient region near a PTV structure even though the effect of grid size on dose accuracy would be relevant to this region (16, 39). This data selection process was not applied to the test dataset used to quantify the network performance. We augmented the training dataset using flip and rotations from −15 to 15° with 5° intervals.

### Network Training and Assessment

Our proposed cascaded networks (HD U-net and RDN) were trained separately using training dataset. The test dataset produced no effects, such as network hyperparameter tuning, during the training process. The HD U-net (first network) was trained with the low-resolution doses (input) and downsampled high-resolution doses (label) for the same dose calculation algorithm. After the downsampled high-resolution dose (input) and the corresponding high-resolution dose planes (label) were converted to percentages, the RDN (second network) was trained using these data regardless of the dose calculation algorithm. We trained three models (defined in *Materials and Methods*. A.), and the trained models were evaluated using the test dataset. The training-evaluation cycle was repeated 5 times using a five-fold cross validation technique. We averaged the outcome from these five repetitions.

The performance assessment focused on the quantification of the spatial/dosimetric parameters and gamma analysis (40) for the low-resolution and predicted high-resolution doses relative to the baseline high-resolution doses. For the spatial assessment, the differences in the dose distributions were created by subtracting the baseline from the low-resolution doses/predicted high-resolution doses, respectively. In addition, the dice similarity coefficient (DSC) values (between the baseline dose and the low-resolution dose/predicted high-resolution dose) were plotted as curves ranging from 0% to 100% isodose volume. For the assessment of dosimetric parameters, first, we calculated dose volume histograms (DVHs) for the PTV and OARs in the baseline, low-resolution doses, and predicted high-resolution dose. The dosimetric parameters, including mean dose, maximum dose, minimum dose, homogeneity index (HI), conformity index (CI), and percentage volume receiving n% of the prescription doses (such as V95% for PTV, V70% for rectum/bladder, V50% for rectum/bladder, and V30% for rectum/bladder), were then analyzed. Three-dimensional global gamma analysis (between the baseline dose and the low-resolution dose/predicted high-resolution dose) was performed with a 2%/2 mm criterion and 10% threshold in PTV, rectum, and bladder. Moreover, statistical analyses were performed for the dosimetric parameters/gamma passing rate using either the paired t-test or Wilcoxon singed rank test after a normality check using the Shapiro-Wilk test.

#### Details of Training

We trained the cascaded networks (HD U-net and RDN, respectively) separately. To train the HD U-net (the first network in the cascaded networks), the low-resolution dose planes (input) and the corresponding downsampled high-resolution dose planes (label) for the same dose calculation algorithm were used. We defined the loss function as the mean square error between the predicted dose (network output) and the label dose planes. At the start of the training process, trainable parameters for the network were initialized by the Glorot uniform initializer (41). The network parameters were optimized by minimizing the loss function using the Adam optimizer (42) with a default setting (β1 = 0.9, β2 = 0.999, ϵ = 1.0e-8). This optimization was performed with a learning rate of 10^-4^ over 100 epochs using mini-batch learning (size = 20). Prior to the training the RDN (second network), the downsampled high-resolution dose (input) and the corresponding high-resolution dose planes (label) were converted to percentages. Then, the RDN was trained using these data, regardless of the dose calculation algorithm. The initialization and optimization of the network parameters were performed following the same procedure as for the HD U-net. The implementations were conducted on a desktop computer (NDVIA Geforce GTX 1050 Ti 4GB) using a framework of TensorFlow 2.0.0 (43). This training was applied to the AAA 3, AAA 5, and AXB 3 models.

#### Spatial Assessment

Dose distribution differences were generated by subtracting the baseline high-resolution doses from the low-resolution doses (which were linearly interpolated to a matrix size consistent with the baseline) and the predicted high-resolution doses, respectively. In addition, to quantify the accuracy, the DSCs for the isodose volume between the baseline and low-resolution doses/predicted doses were calculated according to the following equation:

$$DSC=\frac{2|A\cap B|}{\left|A|+|B\right|}$$

where A and B denote the binary volumes by threshold (≥ arbitrary isodose) for the baseline and other doses, respectively. We plotted the DSC curves ranging from 0 to 100% isodose volumes.

#### Dosimetric Assessment

To quantify the dosimetric accuracy, dose volume histograms for the PTV and OARs (rectum, bladder, left/right femoral heads) were calculated for the baseline high-resolution, predicted high-resolution, and low-resolution doses.

For the PTV, dosimetric parameters, such as the mean dose, maximum dose, minimum dose, and the V95% (the ratio of the percentage of volume irradiated by 95% or more of the prescription dose to the PTV), were calculated. Furthermore, the HI (describing the uniformity of the dose distribution within the target) and CI (the ratio of the reference isodose volume to the PTV) were calculated *via* the following equations:

$$Homogeneity\;index\;(HI)=\frac{D2-D98}{D50}$$

where D2, D98, and D50 denote the doses covering 2%, 98%, and 50% of the PTV, respectively, and

$$Conformity\;index\;(CI)=\frac{V_{RI}}{TV}$$

where V_RI_ denotes the reference isodose volume (set at 95%) for the body, and TV represents the physical volume of the PTV.

For the rectum/bladder, mean, maximum, and minimum doses as well as V_n%_, which is the ratio of the volume irradiated by n% or more of the prescription dose to the OAR volume (set at n = 70, 50, and 30), were evaluated. For left/right femoral heads, mean, maximum, and minimum doses were analyzed.

#### Gamma Analysis

Three-dimensional gamma evaluation was performed to quantitatively compared the baseline high-resolution dose and low-resolution dose/predicted high-resolution dose in the PTV, rectum, bladder. The gamma passing rate was acquired under a dose difference/distance-to-agreement acceptance criterion (2%/2 mm) for global normalization. A low dose threshold was set to 10% of maximum dose (typically used value in clinic).

#### Statistical Analysis

Statistical analyses were performed to evaluate the statistical significance between the baseline high-resolution doses and the low-resolution/predicted high-resolution doses for the dosimetric parameters. In addition, gamma passing rate for low-resolution dose was statistically compared with that for predicted high-resolution dose. The paired t-test or the Wilcoxon signed-rank test was performed using SPSS Statistics 21 (IBM SPSS, Chicago, IL) after a normality check using the Shapiro-Wilk test. The statistical significance was decided as p-value of <0.05.

## Results

This section describes the results for the PTV, bladder, and rectum; the results for other OARs are described in the **Supplementary Data**.

### Computation Time and Training Loss

The mean computation times to predict the high-resolution dose for an axial 2D dose plane were 0.017 s and 0.011 s for the AAA 3/AXB 3 models (from 3 to 1 mm grid) and the AAA 5 model (from 5 to 1 mm grid), respectively.

**Figure 3:**
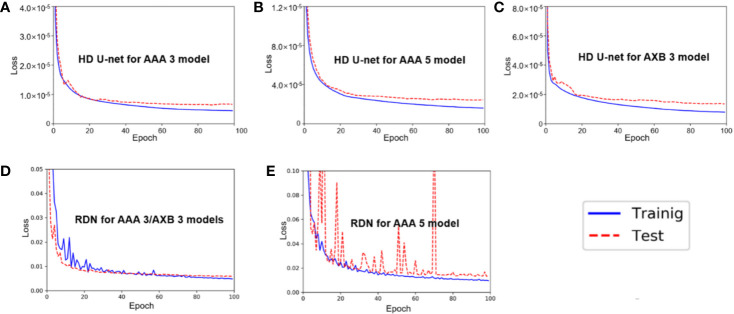
Average training/test loss curves across the five cross-validation folds in **(A)** hierarchically densely connected U-net (HD U-net) for the anisotropic analytical algorithm (AAA) 3 model, **(B)** HD U-net for the AAA 5 model, **(C)** HD U-net for the AXB 3 model, **(D)** residual dense net (RDN) for the AAA 3/AXB 3 models, and **(E)** RDN for the AAA 5 model.

**Figure 3** shows the average training and test loss curves across the five cross-validation folds for each network in three models (AAA 3, AAA 5, and AXB 3). In all networks, the training loss decreased rapidly within 20 epochs, and then slowly after that. A similar trend was observed in the test loss curves; overfitting was not observed in these loss curves.

### Spatial Assessment

**Figure 4:**
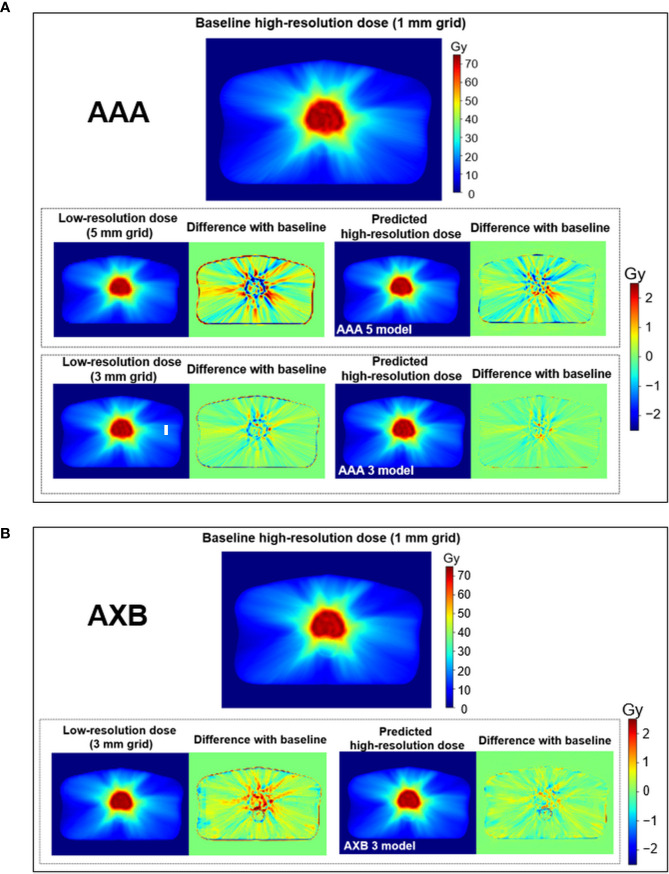
Visual comparison for the low-resolution and predicted high-resolution doses relative to the baseline high-resolution dose at the same axial level in **(A)** the anisotropic analytical algorithm (AAA)-calculated dose and **(B)** the AXB-calculated dose. dose distribution differences were created by subtracting the baseline from the low-resolution/predicted high-resolution doses, respectively. During this process, the low-resolution dose was linearly interpolated to a matrix size equivalent to the baseline. Upper and lower color scale bars in each figure indicate the range of the doses and dose distribution differences, respectively.

**Figure 4** shows the dose distribution differences between the baseline high-resolution dose (1 mm grid) and the low-resolution/predicted high-resolution doses in the same axial level for the AAA and AXB doses. For the AAA dose (**Figure 4A**), the low-resolution dose with a 5 mm grid indicated major errors near the PTV region. Similar errors were observed in the low-resolution dose with a 3 mm grid. The predicted high-resolution dose showed these errors were reduced in both the AAA 3 and AAA 5 models. The predicted dose from the AAA 3 model demonstrated the closest agreement with the baseline. Similar results were observed for the AXB dose (**Figure 4B**).

**Figure 5:**
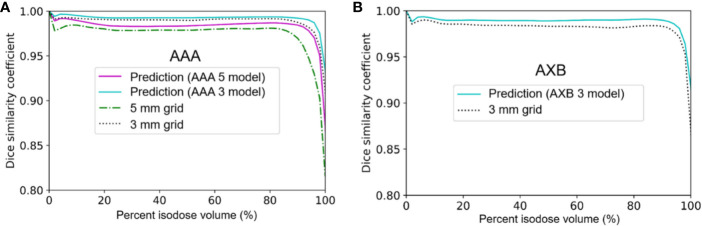
Mean dice similarity coefficient across the five cross-validation folds between the predicted high-resolution and low-resolution doses relative to the baseline high-resolution dose (using a 1 mm grid) in **(A)** the anisotropic analytical algorithm (AAA) dose and **(B)** the Acuros XB (AXB) dose.

**Figure 5** displays the mean DSC plots comparing the percent isodose volume between the baseline dose and the low-resolution/predicted high-resolution doses in the AAA and AXB doses. For the AAA dose (**Figure 5A**), the DSC values for the predicted doses from each model were closer to 1 (ideal value) than those for each low-resolution dose. For the in predicted dose from the AAA 5 model in particular, the DSC values ranging from 80 to 100% isodose volume were dramatically improved. For the AXB dose (**Figure 5B**), the predicted dose indicated higher DSC values compared to the low-resolution dose.

### Dosimetric Evaluation

**Figure 6:**
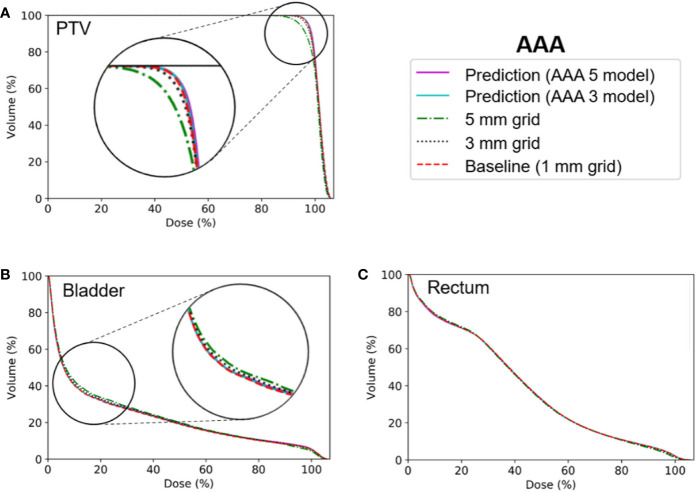
Average dose volume histograms across the five cross-validation folds for the baseline high-resolution anisotropic analytical algorithm (AAA) dose (with a 1 mm grid), the predicted high-resolution AAA dose, and the low-resolution AAA dose (with 3 or 5 mm grids) in **(A)** the planning target volume (PTV), **(B)** the bladder, and **(C)** the rectum. The AAA 3 and AAA 5 models indicate network models for the ×3 super-resolution scale (prediction from a 3 mm to a 1 mm grid) and the ×5 super-resolution scale (from a 5 mm to a 1 mm grid), respectively, for the AAA dose.

**Figure 7:**
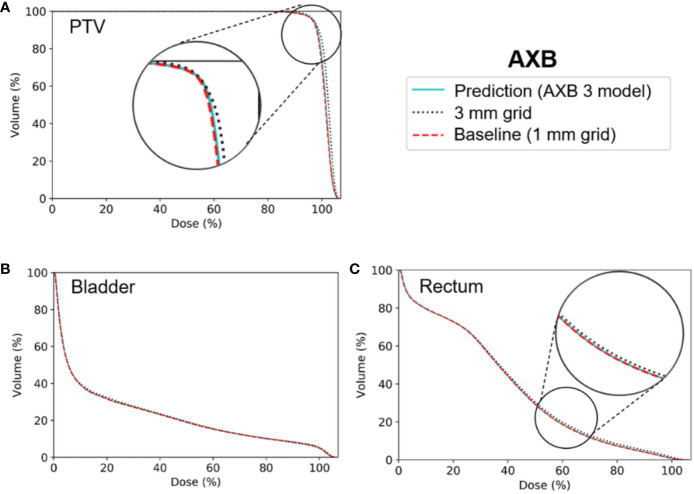
Average dose volume histograms across the five cross-validation folds for the baseline high-resolution Acuros XB (AXB) dose (with a 1 mm grid), the predicted high-resolution AXB dose, and the low-resolution AXB dose (with a 3 grid) in **(A)** the planning target volume (PTV), **(B)** the bladder, and **(C)** the rectum. The AXB 3 model indicates network models for the ×3 super-resolution scale (prediction from a 3 mm to a 1 mm grid) for the AXB dose.

**Figures 6** and **7** show the average dose volume histograms of PTV/OARs for the baseline high-resolution/predicted high-resolution/low-resolution doses in the AAA and AXB doses, respectively. For the AAA dose, the DVHs for the PTV were visually similar to each other, with the exception of the low-resolution dose with a 5 mm grid (**Figure 6A**). For the bladder (**Figure 6B**), a similar trend was observed. In the rectum, as well as the other OARs (left/right femoral heads), differences between the DVHs were not observed. For the AXB dose, the DVH of the PTV for the predicted dose agreed closely with the baseline compared to the low-resolution dose (**Figure 7A**). A similar result was observed for the rectum (**Figure 7C**). In the bladder and the other OARs (left/right femoral heads), there were no differences between the DVH curves.

**Tables 1** and **2** show comparisons of the mean dosimetric parameters between the baseline high-resolution and low-resolution doses/predicted high-resolution doses in the AAA and AXB doses, respectively. For the AAA dose (**Table 1**), the mean dose, maximum dose, V_95%_, and CI for the PTV in the low-resolution doses were underestimated compared to those from the baseline dose. The minimum dose and HI were overestimated. The differences in the dosimetric parameters between the baseline high-resolution dose and the low-resolution dose with a 3 mm grid were 0.3 Gy (mean dose), 2.3 Gy (max. dose), −0.2 Gy (min. dose), −1.0% (V_95%_), −0.01 (HI), and −0.04 (CI), respectively. The corresponding parameter differences for a 5 mm grid were 0.7 Gy (mean dose), 3.5 Gy (max. dose), −0.6 Gy (min. dose), −5.2% (V_95%_), −0.03 (HI), and −0.11 (CI), respectively. By contrast, the corresponding differences for the predicted high-resolution doses for the AAA 3 and AAA 5 models were reduced. These results illustrate that the predicted doses showed a closer agreement with the baseline dose compared to the low-resolution doses. For both the bladder and rectum, the majority of the parameters from the predicted doses demonstrated closer agreement with the baseline doses compared to the low-resolution doses, with similar results observed in the other OARs (left/right femoral heads).

**Table 1:** Comparisons of the mean dosimetric parameters (across the five cross-validation folds) between the baseline high-resolution anisotropic analytical algorithm (AAA) dose (with a 1 mm grid) and the low-resolution AAA dose (with 3 or 5 mm grids)/predicted high-resolution AAA dose in the planning target volume (PTV), bladder, and rectum.

ROI	Parameters	AAA				
		Baseline(1 mm grid)	3 mm grid	Prediction AAA 3 model	5 mm grid	Prediction AAA 5 model
PTV	Mean dose (Gy)	73.3 (4.0)	73.0 (4.1)	**73.3 (4.0)**	72.6 (4.1)	**73.3 (4.0)**
	Max. dose (Gy)	79.7 (3.0)	77.4 (3.7)	79.3 (3.4)	76.2 (4.1)	78.6 (3.8)
	Min. dose (Gy)	61.4 (5.0)	**61.6 (5.3)**	**61.5 (4.9)**	**62.0 (5.1)**	62.9 (3.9)
	V_95%_ (%)	98.9 (0.6)	97.9 (0.9)	99.0 (0.6)	93.7 (3.0)	**98.9 (0.6)**
	CI	1.08 (0.03)	1.04 (0.02)	**1.08 (0.03)**	0.97 (0.04)	**1.07 (0.03)**
	HI	0.09 (0.01)	0.10 (0.01)	0.09 (0.01)	0.12 (0.02)	0.09 (0.01)
Bladder	Mean dose (Gy)	17.1 (11.0)	17.2 (11.1)	17.1 (11.0)	17.4 (11.1)	**17.1 (11.0)**
	Max. dose (Gy)	78.7 (3.2)	76.7 (3.8)	78.1 (3.4)	75.4 (4.2)	77.1 (3.6)
	Min. dose (Gy)	1.1 (1.0)	1.1 (1.0)	1.0 (1.0)	1.2 (1.3)	**1.1 (1.1)**
	V_70%_ (%)	12.9 (11.6)	12.9 (11.5)	**12.9 (11.5)**	**13.0 (11.5)**	**13.1 (11.6)**
	V_50%_ (%)	19.4 (15.9)	19.7 (16.0)	**19.4 (15.9)**	20.0 (16.2)	19.6 (16.0)
	V_30%_ (%)	27.6 (19.6)	28.0 (19.7)	**27.6 (19.6)**	28.6 (20.0)	**27.8 (19.6)**
Rectum	Mean dose (Gy)	28.8 (8.5)	28.7 (8.5)	28.8 (8.5)	28.6 (8.4)	28.7 (8.5)
	Max. dose (Gy)	76.5 (3.5)	75.2 (3.7)	76.0 (3.6)	73.9 (3.9)	75.0 (3.8)
	Min. dose (Gy)	1.0 (0.7)	1.1 (0.7)	1.0 (0.6)	1.2 (0.7)	**1.1 (0.7)**
	V_70%_ (%)	15.4 (7.6)	15.3 (7.5)	**15.4 (7.6)**	**15.3 (7.5)**	15.3 (7.6)
	V_50%_ (%)	32.7 (15.1)	32.4 (15.0)	**32.7 (15.2)**	32.2 (14.6)	**32.6 (15.0)**
	V_30%_ (%)	61.6 (16.0)	61.4 (16.0)	61.5 (16.0)	61.2 (16.0)	61.3 (16.0)

The V_n%_ indicates the percentage of the volume irradiated by n% of the prescription dose. Values expressed in bold are not statistically significant (p-value of >0.05). The standard deviations are indicated in parentheses.

ROI, region of interest; HI, homogeneity index; CI, conformity index; AAA, anisotropic analytical algorithm.

**Table 2:** Comparisons of mean dosimetric parameters (across the five cross-validation folds) between the baseline high-resolution Acuros XB (AXB) dose (with a 1 mm grid) and the low-resolution AXB dose (with a 3 mm grid)/predicted high-resolution AXB dose in the planning target volume (PTV), bladder, and rectum.

ROI	Parameters	AXB		
		Baseline(1 mm grid)	3 mm grid	PredictionAXB 3 model
PTV	Mean dose (Gy)	73.0 (3.7)	73.7 (3.7)	73.1 (3.7)
	Max. dose (Gy)	79.5 (3.3)	79.0 (3.5)	**79.3 (3.7)**
	Min. dose (Gy)	55.3 (4.3)	60.0 (4.3)	56.5 (4.0)
	V_95%_ (%)	97.3 (1.0)	97.8 (0.9)	97.4 (0.9)
	CI	1.04 (0.02)	**1.04 (0.02)**	1.04 (0.02)
	HI	0.11 (0.02)	0.11 (0.01)	0.11 (0.02)
Bladder	Mean dose (Gy)	16.8 (11.0)	17.0 (11.1)	16.8 (10.9)
	Max. dose (Gy)	78.3 (3.2)	**78.0 (3.2)**	77.9 (3.5)
	Min. dose (Gy)	1.0 (0.9)	1.0 (0.9)	1.0 (0.9)
	V_70%_ (%)	12.8 (11.4)	13.0 (11.6)	**12.8 (11.5)**
	V_50%_ (%)	19.2 (15.8)	19.7 (16.0)	**19.3 (15.8)**
	V_30%_ (%)	27.4 (19.4)	27.9 (19.7)	**27.4 (19.4)**
Rectum	Mean dose (Gy)	27.3 (8.0)	27.9 (8.1)	**27.3 (8.0)**
	Max. dose (Gy)	75.2 (4.2)	**75.3 (4.0)**	74.9 (4.0)
	Min. dose (Gy)	1.0 (0.6)	1.0 (0.6)	1.0 (0.6)
	V_70%_ (%)	11.8 (6.6)	12.9 (6.8)	11.9 (6.5)
	V_50%_ (%)	29.2 (14.4)	30.3 (14.4)	**29.2 (14.3)**
	V_30%_ (%)	60.8 (16.8)	61.3 (16.5)	**60.8 (16.7)**

The V_n%_ indicates the percentage of the volume irradiated by n% of the prescription dose. Values expressed in bold are not statistically significant (p-value of >0.05). The standard deviations are indicated in parentheses.

ROI, region of interest; HI, homogeneity index; CI, conformity index; AXB, Acuros XB.

Among the dosimetric parameters for the AXB dose (**Table 2**), the minimum dose for the PTV from the low-resolution dose for a 3 mm grid was the most overestimated value compared to the corresponding value from the baseline dose; the difference for the minimum dose between the baseline and low-resolution dose was −4.7 Gy. In the predicted dose, the difference was reduced to −1.2 Gy. Moreover, the remaining dosimetric parameters for the PTV in the predicted dose were observed more closely to those in the baseline dose compared to the low-resolution dose. For both the bladder and the rectum, the parameters from the predicted doses were more similar to those from the baseline than low-resolution doses, with the exception of the maximum dose. Similar results were obtained in the left/right femoral heads.

### Gamma Evaluation

**Table 3** shows comparisons of the mean gamma passing rates for low-resolution and predicted high-resolution doses in the AAA/AXB doses. The gamma passing rates for the predicted high-resolution doses from AAA 3, AAA 5, and AXB 3 models were substantially improved compared to those for the low-resolution doses in all region of interests (PTV, bladder, and rectum). These improvements were statistically significant (p-values of<0.05). Especially the gamma passing rates for the predicted high-resolution doses except for AAA 5 model were higher than 98%, although those for the low-resolution doses in the PTV were lower than 95%. The high-resolution dose from AAA 5 model showed lower gamma passing rate than that from AAA 3 and AXB 3 models.

**Table 3:** Comparisons of mean gamma passing rates (across the five cross-validation folds) for low-resolution dose (with 3 mm or 5 mm grids) and predicted high-resolution dose in the planning target volume (PTV), bladder, and rectum.

ROI	Gamma passing rate with a 2%/2 mm criterion (%)					
	AAA				AXB	
	3 mm grid	Prediction AAA 3 model	5 mm grid	Prediction AAA 5 model	3 mm grid	Prediction AXB 3 model
PTV	93.6 (3.7)	99.1 (1.0)	71.0 (5.8)	92.4 (4.6)	88.0 (6.2)	98.2 (2.6)
Bladder	98.1 (2.1)	99.5 (1.3)	90.0 (4.7)	97.9 (2.3)	98.0 (2.1)	99.1 (2.4)
Rectum	98.8 (0.8)	100.0 (0.1)	72.4 (6.9)	99.0 (1.1)	98.7 (0.6)	99.4 (0.4)

The standard deviations are indicated in parentheses.

ROI, region of interest; AAA, anisotropic analytical algorithm; AXB, Acuros XB.

### Statistical Analysis

Comparing the baseline AAA doses (with a 1 mm grid) with the low-resolution AAA doses (with a 3 mm grid), the p-values for most parameters were less than 0.01. This indicates that there are statistically significant differences between the two doses except for a few parameters (such as the minimum dose for PTV and V_70%_ for the bladder/rectum). By contrast, no significant differences (p-values of >0.05) were observed between the baseline and the predicted high-resolution dose (AAA 3 model) for the majority of parameters (**Table 1**). Likewise, a similar trend was observed for the predicted high-resolution doses for the AAA 5 and AXB 3 models, as shown in **Tables 1** and **2**.

## Discussion

This paper is the first to attempt the prediction for high-resolution dose with a 1 mm grid using the cascaded networks in clinical prostate VMAT. We have presented a detailed outline of the network construction/data preparation/training procedure and quantified the network performance, including spatial/dosimetric parameters. Our model consisted of two cascaded networks (HD U-net and RDN), which were trained separately. The proposed model took <0.02 s to predict one axial high-resolution dose plane from the low-resolution dose plane (with 3 or 5 mm grids) for the same dose calculation algorithm. Compared to low-resolution doses, the predicted high-resolution doses were visually similar to the baseline high-resolution doses; furthermore, the DSC values of the predicted doses were closer to 1 than those of the low-resolution doses. The DVH curves for the PTV/OARs for the predicted dose were more consistent with the DVH curves for the baseline than the low-resolution dose. For the predicted dose, the average of the dosimetric parameters, including mean dose, maximum dose, minimum dose, HI, CI, V95%, V70%, V50%, and V30% were closer to corresponding parameters obtained in the baseline than the low-resolution dose. Moreover, the majority of these parameters demonstrated no statistically significant differences between the baseline and predicted doses. The gamma passing rates based on a 2%/2 mm criterion for the predicted high-resolution does were higher than those for the low-resolution doses. These results indicate that the proposed network is capable of achieving dose super-resolution with reduced computation time.

The AAA 5 model (prediction of the high-resolution AAA dose from the low-resolution AAA dose with a 5 mm grid) showed poor performance when compared to the AAA 3 model (prediction from the low-resolution AAA dose with a 3 mm grid). One reason for this is that the quality of the input for the AAA 5 model was relatively poor compared to the AAA 3 model. Another reason is the large super-resolution scale (×5 for the AAA 5 model vs. ×3 for the AAA 3 model), which can cause a blurring effect in the predicted dose. Similar observations were recorded by previous studies exploring image super-resolution in the field of computer vision (36, 44, 45). Despite these problems, the predicted high-resolution doses for the AAA 5 model were better agreement with the baseline high-resolution doses than the low-resolution AAA doses (with 3 and 5 mm grids). For the AXB 3 model (prediction from the low-resolution AXB dose with a 3 mm grid), it was observed slightly lower performance compared to the AAA 3 model. This may be because bicubic downsampling of the high-resolution doses (for training dataset of the HD U-net). The downsampling cause a smoothing effect (46), which does not completely preserve characteristics of the high-resolution doses. Especially, this issue might be more pronounced in the downsampled high-resolution AXB doses than in the downsampled high-resolution AAA doses because of a heterogeneity correction. Despite the issue, the AXB model showed higher accuracy than the low-resolution AXB doses with 3 mm grid as shown in **Tables 2** and **3**.

Our study focused on achieving dose super-resolution for the same dose calculation algorithm, as opposed to the conversion of the AAA-calculated doses to the AXB-calculated doses. Obviously, converting the AAA-calculated dose with a 5 mm grid to that for the AXB algorithm with a 1 mm grid has a great advantage for computation time; this is because the AAA with a 5 mm grid requires the shortest time for dose calculation, whereas the AXB algorithm with a 1 mm grid requires the longest time [a time difference of about 3931 s in a prostate VMAT plan, see Kim et al. (22)]. However, one drawback associated with this process is that this conversion requires not only the dose data but also additional information such as CT images (34, 35). The need for this additional information may cause inconvenience to user. Moreover, difference in calculation time between the AAA with a 5 mm grid and the AXB algorithm with a 3 mm grid was about 132 s for prostate VMAT plans (22), which exist within a clinically acceptable calculation time frame. These support that our dose super-resolution approach in the same dose calculation algorithm (i.e., converting the AXB-calculated dose with a 3 mm grid to that with a 1 mm grid) is sufficiently useful.

Our proposed method combined some advantages of previously reported networks (27, 38, 47). The first is the use of residual dense learning (27, 47), which reduces the network complexity by reducing the number of trainable parameters compared to the original U-net. The second is the use of sub-pixel convolution (known as ESPCN) (38) to recover high-resolution dose planes. This reduces computation time greatly by rearranging feature maps of the network to high-resolution frame. The advantages of our network facilitated shorter computation times (<0.02 s for one axial dose plane) compared to previously proposed network (0.2 s for one axial dose plane) by Sumida et al. (35) despite achieving dose super-resolution for a finer grid (1 vs. 2 mm) with lower graphics processing unit power (GTX 1050 Ti 4GB vs. GTX 1060 6GB).

Although this study focused on prostate VMAT plans only, retraining the network will likely enable the proposed method to be applied to other sites, such as the lungs. For example, a previous study related to lung VMAT recommended using the AXB algorithm with a 2.5 mm grid instead of a 1 mm grid due to clinically acceptable calculation time despite the higher accuracy offered by the 1 mm grid (48). Our method would improve this problem. Particularly, in the lungs with high heterogeneity, the dose super-resolution method may be more useful. Our AXB 3 model showed good performance in the rectum (heterogeneous region), which indicates it has the potential to be sufficiently applied to lungs. Therefore, potential future work includes application of such lung VMAT plans. In addition, the dose super-resolution method could potentially apply to adaptive radiation therapy to accurately predict daily dose distribution.

Our dose super-resolution method can be applied in clinic as a useful tool to assist in improvement of treatment planning. For example, patient cases with noticeable errors in dose calculations by large grid sizes can be quickly identified through subtraction between calculated low-resolution dose and predicted high-resolution dose. For these cases only, the dose distributions can be re-calculated with a 1 mm grid. In other words, our method can provide the radiotherapy staff to information on cases that require dose calculation with a small grid size.

The proposed method has some limitations. One of these limitations is the small dataset (73 patients). To mitigate the effects (such as overfitting) relating to the small dataset, we used k-fold cross validation (set at k = 5) and data augmentation (flip and rotations), which are widely recognized as appropriate techniques for reducing overfitting (49, 50). Furthermore, the dense connections used in our network architecture have been reported to decrease the likelihood of overfitting, even with a small dataset, through the regularization effect (47). While no overfitting was observed in our networks (**Figure 3**), a larger dataset is required to improve the network performance. A second limitation is that the network is based on 2D dose planes, which could lead to some errors at the superior and inferior edges of the high dose volume regions. This limitation also applies to previous studies focusing on dose super-resolution due to mainly memory shortage (34, 35). In the field of medical image segmentation, various networks such as V-net (51) and 3D U-net (52) have been proposed, which used 3D convolution efficiently. Moreover, while the HD U-net used in this study was constructed using 2D operations (such as 2D convolution) due to memory shortage, the original HD U-net (27) was based on 3D operations to predict 3D dose volume from the PTV, OAR structures, and prescription dose. Therefore, future work will focus on a deep learning model for dose super-resolution based on the 3D dose volume.

## Conclusion

We propose a cascaded network model that exhibits performance similar to the dose super-resolution of a 1 mm grid with reduced computation time in prostate VMAT. Our findings indicate a good agreement between baseline high-resolution doses with a 1 mm grid and predicted high-resolution doses. Our dose super-resolution approach uses only dose distributions without additional data such as CT images, which provides advantages of convenience to the user over other dose super-resolution methods. Therefore, our model could be easily applied to the clinic. The proposed method demonstrates immense potential and can be extended easily to other sites, such as the lungs, through retraining the network. In addition, our model could be a useful tool in other radiotherapy technique such as adaptive radiation therapy.

## Data Availability Statement

The raw data supporting the conclusions of this article will be made available by the authors, without undue reservation.

## Ethics Statement

The studies involving human participants were reviewed and approved by Seoul National Bundang Hospital Institutional Review Board Protocol (B-2004/608-112) for retrospective 73 cases. The patients/participants provided their written informed consent to participate in this study.

## Author Contributions

Study concept and design: D-SS, TS, and J-BC. Data acquisition: D-SS, K-HK, J-SK, and J-BC. Quality control of data: S-WK, S-HK, and J-SK. Network construction and training: D-SS, K-HK, and S-WK. Data analysis and interpretation: D-SS, K-HK, S-WK, S-HK, T-HK, D-SK, WC, TS, and J-BC. Manuscript preparation and editing: D-SS and J-BC. All authors contributed to the article and approved the submitted version.

## Funding

This research was supported by the Mid-career Researcher Program (Grant No. 2018R1A2B2005343) through the National Research Foundation of Korea and by the Seoul National University Bundang Hospital (SNUBH) Research Fund (Grant No. 13-2018-013).

## Conflict of Interest

The authors declare that the research was conducted in the absence of any commercial or financial relationships that could be construed as a potential conflict of interest.
