# Supplementary material for: Dose Super-Resolution in Prostate Volumetric Modulated Arc Therapy Using Cascaded Deep Learning Networks
Source: Front Oncol. 2020 Nov 16;10:593381. doi: 10.3389/fonc.2020.593381 (PMC7701297; doi:10.3389/fonc.2020.593381)
Supplement: Supplementary file 1 [file DataSheet_1.docx]

Supplementary Material

# Supplementary Figures


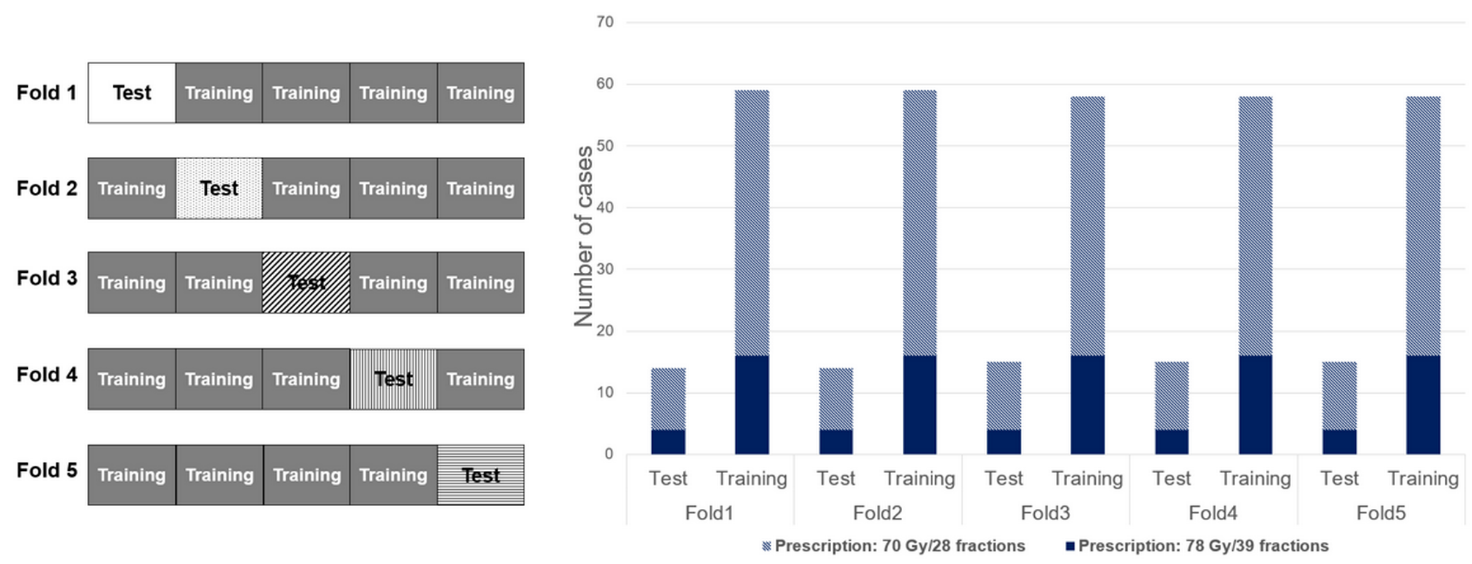


Figure S1. Distribution of dataset.


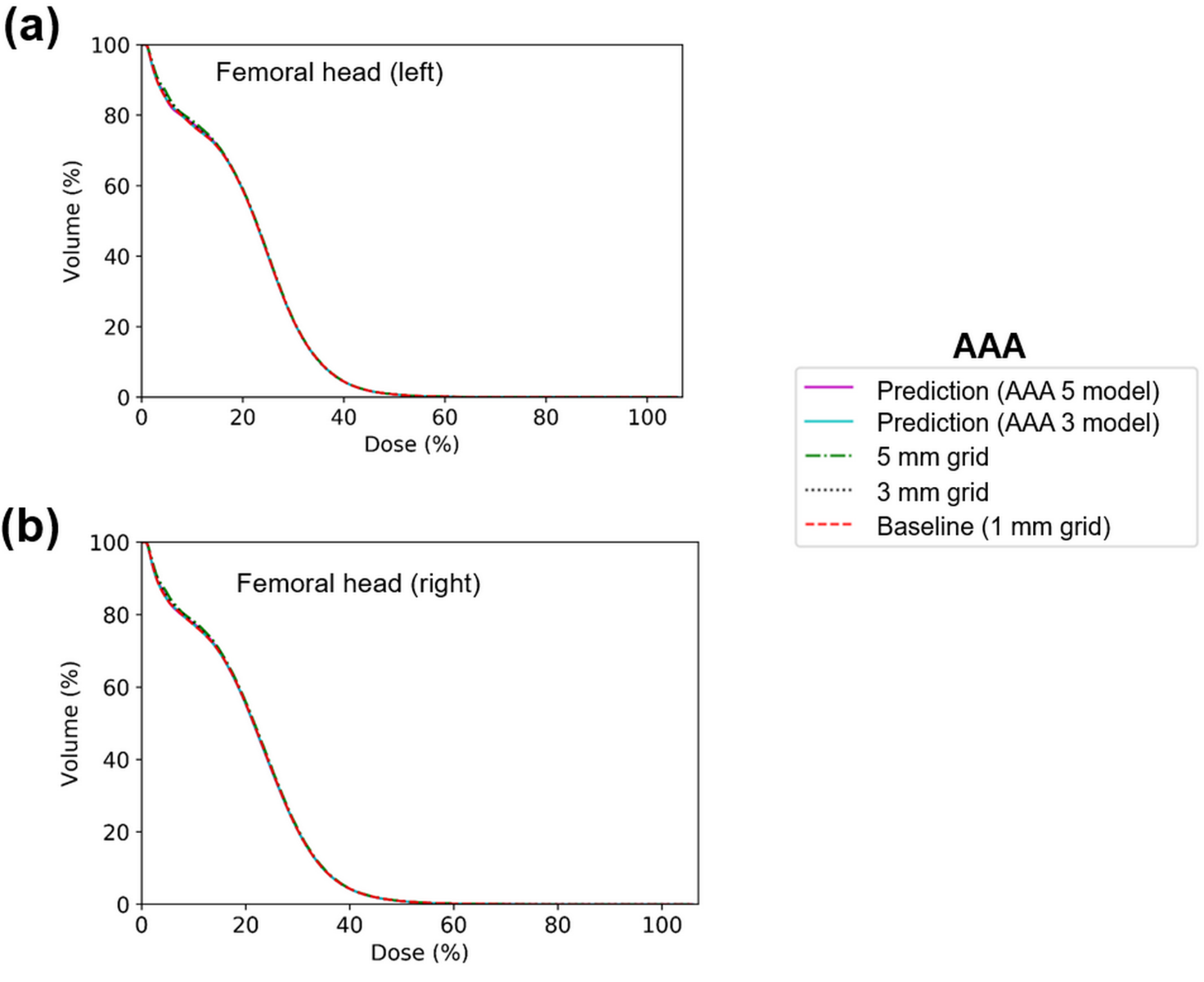


Figure S2. Average dose volume histograms across the five cross-validation folds for the baseline high-resolution AAA dose (with a 1 mm grid), the predicted high-resolution AAA dose, and the low-resolution AAA dose (with 3 or 5 mm grids) in (a) the left femoral head and (b) the right femoral head.


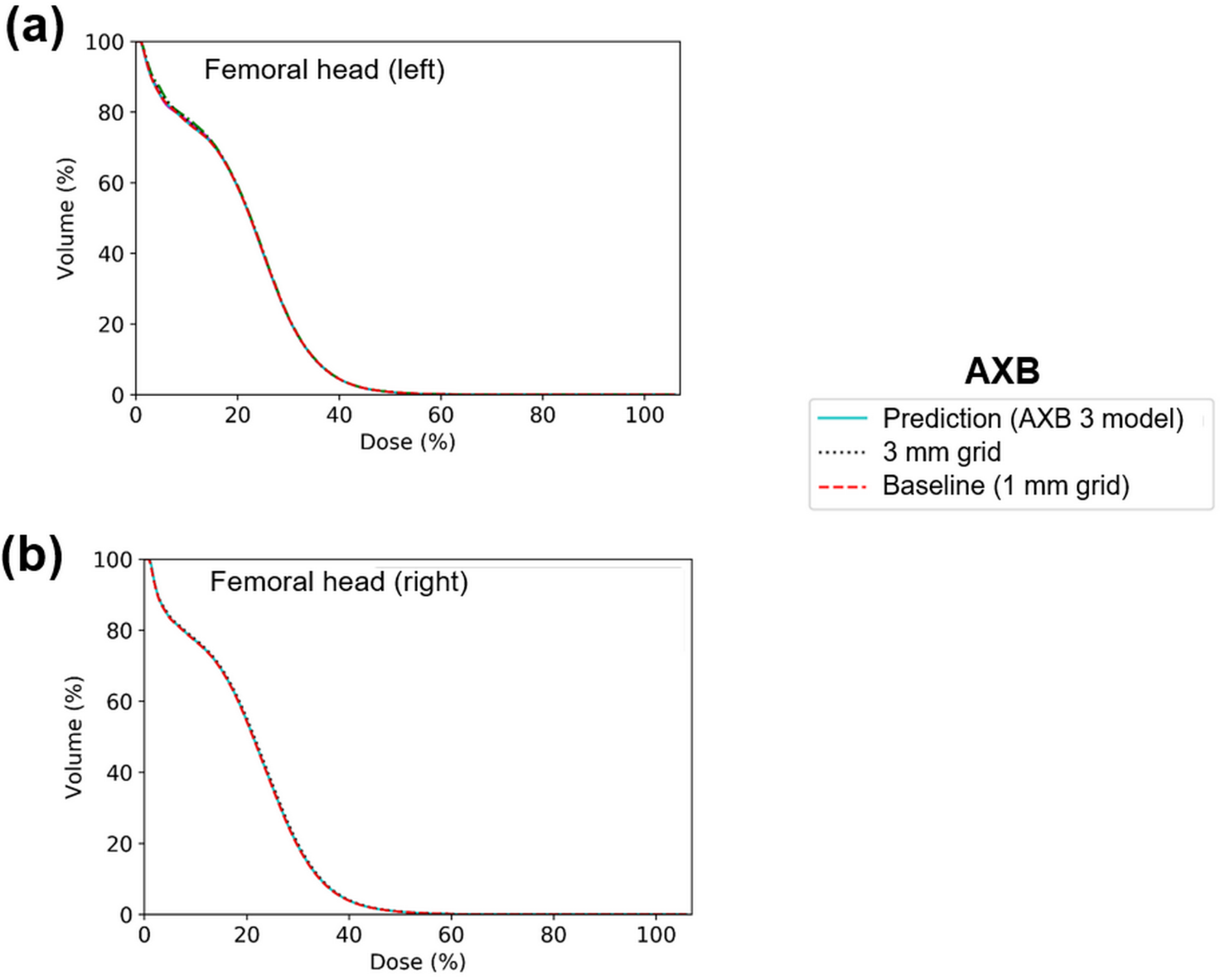


Figure S3. Average dose volume histograms across the five cross-validation folds for the baseline high-resolution AXB dose (with a 1 mm grid), the predicted high-resolution AXB dose, and the low-resolution AXB dose (with a 3 mm grid) in (a) the left femoral head and (b) the right femoral head.

# Supplementary Tables

| ROI | Parameters | AAA | | | | |
| --- | --- | --- | --- | --- | --- | --- |
|  |  | Baseline  (1 mm grid) | 3 mm grid | Prediction AAA 3 model | 5 mm grid | Prediction AAA 5 model |
| Left femoral head | Mean dose (Gy) | 15.3 (6.0) | 15.3 (6.0) | **15.3 (6.0)** | 15.4 (6.0) | **15.3 (6.0)** |
|  | Max. dose (Gy) | 30.6 (7.8) | 30.0 (7.5) | 30.5 (7.7) | 29.2 (7.1) | 30.1 (7.7) |
|  | Min. dose (Gy) | 2.6 (2.9) | 2.7 (3.0) | 2.6 (2.9) | 3.1 (3.3) | **2.8 (3.1)** |
| Right femoral head | Mean dose (Gy) | 15.0 (6.5) | 15.0 (6.5) | 14.9 (6.5) | 15.1 (6.5) | **15.0 (6.5)** |
|  | Max. dose (Gy) | 30.0 (8.0) | 29.4 (7.8) | 29.9 (8.0) | 28.5 (7.9) | 29.4 (7.9) |
|  | Min. dose (Gy) | 2.9 (3.2) | 3.0 (3.4) | 2.9 (3.3) | 3.6 (3.8) | 3.2 (3.6) |

Table S1. Comparisons of mean dosimetric parameters, such as the mean, maximum, and minimum doses, (across the five cross-validation folds) between the baseline high-resolution AAA dose (1 mm grid) and the low-resolution AAA dose (3 or 5 mm grids)/predicted high-resolution AAA dose for the left/right femoral heads. Values expressed in bold are not statistically significant (p-value of >0.05). The standard deviations are indicated in parentheses.

Abbreviations: ROI = region of interest; AAA = anisotropic analytical algorithm

| ROI | Parameters | AXB | | |
| --- | --- | --- | --- | --- |
|  |  | Baseline  (1 mm grid) | 3 mm grid | Prediction  AXB 3 model |
| Left femoral head | Mean dose (Gy) | 15.0 (5.9) | 15.2 (5.9) | 15.0 (5.9) |
|  | Max. dose (Gy) | 30.3 (7.5) | 29.8 (7.3) | **30.2 (7.5)** |
|  | Min. dose (Gy) | 2.5 (2.9) | 2.6 (3.0) | **2.5 (2.9)** |
| Right femoral head | Mean dose (Gy) | 14.7 (6.3) | 14.9 (6.4) | **14.7 (6.4)** |
|  | Max. dose (Gy) | 29.8 (7.9) | 29.4 (7.8) | 29.6 (7.8) |
|  | Min. dose (Gy) | 2.8 (3.1) | 2.9 (3.3) | 2.8 (3.1) |

Table S2. Comparisons of mean dosimetric parameters, such as mean, maximum, and minimum doses, (across the five cross-validation folds) between the baseline high-resolution AXB dose (1 mm grid) and low-resolution AXB dose (3 mm grid)/predicted high-resolution AXB dose for the left/right femoral heads. Values expressed in bold are not statistically significant (p-value of >0.05). The standard deviations are indicated in parentheses.

Abbreviations: ROI = region of interest; AXB = Acuros XB
